# Supplementary material for: Are carbon emissions trading and green financial instruments synergistic? -Comprehensive quantitative research based on content analysis
Source: PLoS One. 2024 Mar 7;19(3):e0298601. doi: 10.1371/journal.pone.0298601 (PMC10919601; doi:10.1371/journal.pone.0298601)
Supplement: S1 Data — (ZIP) [file pone.0298601.s001.zip › Data/地级市Z_F工作报告.zip!/░▓╗╒/░▓╟∞╩╨/└┤╫╘╣1⁄2╓┌║┼í╛┬φ┐╦╩2╛▌═°í┐_2014.docx]

政府工作报告 
——2014年1月21日在安庆市第十六届人民代表大会第三次会议上
市长 魏晓明

各位代表：

　　现在，我代表市人民政府，向大会报告政府工作，请予审议，并请列席会议的市政协委员和其他人员提出意见。

　　一、2013年工作回顾

　　刚刚过去的2013年，是本届政府的开局之年。全市上下在中共安庆市委的坚强领导下，深入贯彻落实党的十八大和十八届二中、三中全会精神，坚持稳中求进，统筹稳增长、调结构、促改革、惠民生，开拓进取，奋发有为，圆满完成市十六届人大一次会议确定的主要目标任务，发展成绩令人瞩目。

　　(一)经济发展提速增效。出台工业、现代服务业、现代农业、自主创新等促进政策，扶持实体经济发展。主要指标增速超过全省平均水平、位次好于上年，预计地区生产总值增长11%左右，社会消费品零售总额增长13.5%，固定资产投资首次过千亿、增长20%以上，财政收入197亿元、增长15.7%。经济运行质量明显提升，前11个月工业利润增长46.8%，全年非税收入占比下降1.9个百分点。工业经济快速增长。石化800万吨炼化一体化、华茂佰斯特2000万米面料、卫康制药标准化生产基地等项目建成投产，安庆电厂2×100万千瓦超超临界发电、曙光化工煤制氢、飞凯高分子3千吨电子级超纯氧化铝、丹凤5万吨电子纱及1.5亿米基布等项目加快建设，中安华谊新材料、万博丰5万吨苯甲酸、易昌泰模具等一批项目开工。完成工业投资640亿元，工业用电量增长8.9%，新增规上企业151家，规上工业增加值增长14.4%。科技创新得到加强。实施科技创新“一招鲜”工程，合肥国家大学科技园安庆园运营，东南大学国家技术转移(安庆)中心落成，国家石化质检(安庆)中心挂牌，国家高分子材料质检(桐城)中心建成。新增高新技术企业28家，专利申请、发明专利增幅均居全省第一。战略性新兴产业产值增长21.9%，占工业比重10%。新增中国驰名商标10件，增量全省第一。新增安徽名牌产品14个，总量全省第二。现代服务业加快发展。金融机构存款余额1980.6亿元，贷款余额首次突破千亿，贷款总量和增量均居全省第三。市级投融资平台整合力度加大。招商银行入驻，安粮期货在宜营业，新组建村镇银行2家。房地产投资增长51.5%，销售面积增长43.9%。我市入选国家物流园区布局城市，一类口岸核心能力建设通过验收。迎江区成为全省服务业综合改革试点，潜山县列入全省农村商品流通体系建设试点。“中国网库”安庆平台投入运营，淘宝网“特色中国·安庆馆”启动建设。公共保税仓库建成。外贸进出口总额18亿美元，增长45.7%，增幅全省第二。服务业比重提高到30.1%。农业产业化稳步提升。夏粮八连增，粮食总产247.6万吨。获批国家农业科技园区，新增省级现代农业示范区4个。建成高标准基本农田53.2万亩。农业规模经营165万亩，耕地流转率全省第二。新增省级农业产业化龙头企业39家，增量、总量均居全省第一。新增农民专业合作社1144家、家庭农场1072个。岳西翠兰成为国家地理标志保护产品，桐城小花、枞阳媒鸭获得国家地理标志证明商标。园区发展、招商引资成效明显。省级高新区创建成功，桐城开发区晋升国家级，宜秀都市产业园开工，华茂枞阳工业园、望江童装产业园加快建设。建成标准化厂房111.6万平方米。出台招商导则，权威机构毕马威公司编制的《安庆投资环境评估报告》发布。推进产业招商、驻外招商，香港、广州、上海招商活动取得实效。与央企、知名民企合作加强，与世界500强美国卡特彼勒公司合作的马克柴油机项目投产。全年利用市外资金1185.2亿元，增幅全省第一。

　　(二)城乡建设步伐加快。城市建设快速推进。城镇建设完成投资223.3亿元，市区市政公用设施投资是前三年的总和。建成环城南路、柘山路体育中心段，完成湖心南路、菱湖北路西段、菱湖风景区环湖绿道、39个渠化路口、8个交通环岛改造，人民路综合改造、康熙河(新河)整治、市体育中心等工程扎实推进，骨干道路绿化提升工程实施，54条背街后巷整治基本完工。220千伏迎江输变电工程投入使用。花亭大沟治理、大湖补水一期工程、顺安河泵站汛前完成。特色县城、风情小镇建设加强，城镇承载力进一步增强。城镇化率47.8%，提高1.9个百分点。城市管理力度加大。大力推进省级文明城市创建工作，市区环卫保洁市场化改革进一步深化，交通秩序和户外广告整治初见成效，建筑垃圾处置逐步规范，市容环境明显改善。土地“三项清理”取得实效，我市纳入全国工矿废弃地和低丘缓坡地开发利用试点。交通建设得到加强。安庆长江铁路大桥主体完工。岳武高速、望东长江大桥及北岸连接线工程快速推进。大宜城快速通道工程有序进展。集贤关收费站提前撤除。改造提升国省道130公里、县乡道188公里。石门湖航道疏浚稳步推进，安庆电厂码头投入运行。民航客流量突破11万人次。美好乡村建设全面展开。完成85个重点示范村建设任务，整治1000个自然村，改造3万户农村危房，实施36个乡镇农村清洁工程，除险加固31座病险水库，改造161座农村公路危桥，解决23.6万农村人口饮水安全问题，“三线三边”环境整治启动实施。潜山成为国家美好乡村综合标准化示范县，宿松县洲头乡入选全国美丽乡村创建试点。

　　(三)文化强市全面推进。文化事业繁荣发展。我市获准创建国家公共文化服务体系示范区，乡镇综合文化站、农家书屋实现全覆盖，公共文化场馆全面免费开放，数字图书馆推广工程启动实施，新增6个国家一级图书馆，市少儿图书馆异址开放。新增陈独秀墓、安徽大学红楼及敬敷书院旧址等11处全国重点文物保护单位，新增52处省级文物保护单位。第二轮修志全面完成。创新举办“黄金周·黄梅戏”展演活动，黄梅戏“唱起来、走下去、走天下”成为共识。《半个月亮》荣获中国戏剧节优秀导演奖、表演奖和剧目奖，主演王琴获得“梅花奖”，《徽州往事》获“十艺节”文华剧目奖。文化旅游产业融合发展。全市整体纳入皖南国际文化旅游示范区。我市第一部原创动漫片《宜城小妹》播出。再芬股份公司上市扎实推进，10家企业进入全省民营文化企业百强。五千年文博园二期工程、中国桐城文化博物馆等项目快速推进，“长江旅游从安庆起航”项目落地，新增4A旅游景区3家。“千年古宜城、百景新安庆”人气提升，游客接待量增长12.6%，旅游总收入增长12.9%。

　　(四)生态建设力度加大。“创模”全面启动。蓝天、碧水等七大工程启动实施，47个“创模”项目开工建设。市区建成污水管网56公里，城市地表水治理、西小湖水质生态修复工程进展顺利，石化新装置安全卫生防护范围拆迁工作基本完成。岳西创建国家生态县成效明显。“创森”通过验收。全市森林覆盖率37.1%、林木绿化率39.9%，市区绿化覆盖率40.9%、人均公共绿地面积11.3平方米，桐城、岳西创建省级森林城市通过核查。百万亩森林增长提质工程任务完成近三分之一，“青山白化”整治工作有序推进。节能减排任务完成。淘汰水泥落后产能30万吨，关闭非煤矿山和造纸、粘土砖企业151家。万元工业增加值综合能耗下降5%。

　　(五)人民生活明显改善。33项民生工程任务全面完成。城乡居民收入倍增规划深入实施，城镇居民人均可支配收入增长9%，农民人均纯收入增长14%。居民消费价格指数低于全年控价目标。新增城镇就业9.6万人、下岗失业人员再就业2.6万人，城镇登记失业率控制在4.2%以内。社会保险基金市级统筹有序推进，医疗保险异地结算进程加快。城市低保标准提高12.3%，农村低保标准提高19.9%。计划生育家庭奖励扶助37610人。开展社区居家养老服务试点，新增农村五保供养床位2500张。开工建设保障性住房2.7万套、基本建成1.8万套，市区建设还建房340万平方米。住房公积金制度覆盖面进一步扩大。整治菜市场、早餐摊点、夜市排档和豆腐作坊，便民服务“五放心”体系建设加强。在全省率先建成市级残疾儿童综合康复服务中心。推进专项扶贫、行业扶贫和社会扶贫，实施168个扶贫开发“整村推进”工程，减少10万贫困人口。

      (六)社会建设扎实有效。我市成为全省唯一的国家中小学教育质量综合评价改革实验区，新建、改扩建幼儿园506所，市区首次实现公办义务教育“零择校”，与此同时，6300多名进城务工人员随迁子女免试就近入学，新增省级示范高中2所，安庆二中碧桂园分校投入使用。大力化解高校债务，组建职业教育联盟，促进高等教育服务地方发展。县级公立医院改革扎实推进，医药分开启动实施，市立医院新院区建设加快。食品药品监管加强，禽流感防控扎实有效。省运会体育场馆建设进展顺利，市区51所公办学校体育场馆面向社会开放。坚持领导干部开门接访、带案下访，信访积案化解工作加强。预防和解决农民工工资拖欠机制进一步健全。“安全生产建设年”活动成效明显，煤矿和烟花爆竹生产企业全面退出，事故起数、死亡人数大幅度下降。应急管理得到加强。社会治安综合治理取得实效，治安、刑事案件发案率下降、破案率上升。残疾人、老年人和妇女、儿童权益得到保障，民族宗教、外事侨务工作切实加强。连续7次获得省级“双拥模范城”称号，加强县（市）区人武部建设做法全省推广，陆军预备役步兵二团、宜秀区人武部成为全省标兵单位。国防动员、人民防空、国家安全、地震、气象、涉台、保密、档案、红十字、关心下一代等工作取得新成绩。

　　一年来，我们高度重视政府自身建设，主动接受人大、政协及社会各界监督，办结市人大代表议案建议148件、市政协提案436件，办结率100%。全面推行电子政务，加强政府网站建设，政务公开工作全省领先。建立政府法律顾问制度，完善学法制度，法治政府建设加强。土地、规划、国资、城建、重大项目、投融资机制逐步健全，招投标和公共资源交易进一步规范，建设管理“六分开”、公益性公用工程“四到位”启动实施。着力打造“三最”城市，行政审批项目从280项降到145项，实行企业注册登记“一表通”制度。免除园区工业项目行政事业性收费42项，79项经营服务性收费取消7项、降标35项。电视“百姓问政”、“1584”政风行风热线深受群众好评。严格执行改进工作作风、密切联系群众各项规定，深入开展廉政风险防控，加大行政监察和审计监督力度，全市各级查办违法违纪案件534件、处分547人。

　　各位代表、各位委员、各位同志，一年的工作历历在目，发展的成绩鼓舞人心。这是省委省政府和市委坚强领导的结果，是市人大、市政协和社会各界监督支持的结果，是全市人民共同奋斗的结果。在此，我代表市人民政府，向全市广大工人、农民、知识分子、干部、驻宜解放军指战员、武警消防官兵和政法干警，向各民主党派、各人民团体和社会各界人士，向关心支持安庆建设发展的中央和省驻宜单位、海内外友好人士，向在我市创业的投资者、建设者，表示崇高的敬意和衷心的感谢！

　　在充分肯定成绩的同时，我们清醒地认识到发展中还存在不少困难和问题：整体经济实力还不够强，中心城市辐射带动力不足，县域经济发展还不够快；开放程度不高，新产业、新业态需要大力引进和培育；生态建设和环境保护压力比较大；公共服务和社会保障水平与群众需求存在差距，社会建设和管理还要加强；政府职能转变必须深化，改革创新需要大力推进；干部思想观念、能力本领、工作作风与科学发展要求还不很适应。对此，我们一定认真对待，逐步解决。

　　二、2014年重点工作

　　今年是贯彻落实党的十八届三中全会精神、全面深化改革的第一年，也是完成“十二五”规划目标任务的关键一年。工作的总体要求是：全面贯彻落实党的十八大、十八届二中和三中全会、中央经济工作会议精神，按照省委省政府和市委的决策部署，坚持稳中求进、改革创新，优化发展环境，增强发展活力，转变发展方式，着力调整经济结构，扎实推进新型工业化和城镇化，大力加强公共服务体系建设，促进经济较快发展、民生持续改善，加快经济强市、文化强市、生态强市建设进程。

　　综合考虑，今年全市经济社会发展的主要预期目标是：地区生产总值增长10.5%，财政收入增长11%，社会消费品零售总额增长13%，固定资产投资增长20%，城镇居民人均可支配收入增长10.5%，农民人均纯收入增长11.5%，城镇登记失业率控制在4.2%以内，人口自然增长率控制在6.5％。以内。确保完成节能减排任务。

　　重点做好八个方面工作：

      (一)大力推进新型工业化。实现安庆又好又快发展，必须坚定不移推进工业强市。

　   加快工业转型升级。启动工业腾飞“2135”三年行动计划，力争农产品精深加工产业产值今年过千亿，到2016年，石油化工产业突破千亿，纺织服装、装备制造、战略性新兴产业均超500亿。今年实施150个亿元以上重点工业投资改造项目，完成投资750亿元，力争规上企业增加200家以上。推进石化1000万吨炼油完善工程，加快中安华谊新材料、曙光化工园建设，推动石化产业向精细化工延伸。支持华茂“走出去、引进来”，鼓励申洲针织做大做强，促进纺织服装产业加快发展。扶持环新、恒昌机械、西锐重工发展壮大，促进装备制造业由零部件向整机跨越。大力支持新能源、新材料、智能制造、电子信息等高端产业发展，加快推进安庆电厂二期工程。发挥长江水运优势，打造临江产业集群，支持华泰林浆纸做优做强，推进船用装备生产基地建设。大力引进行业龙头企业，积极支持本地企业通过并购重组等途径加快发展。新建500万平方米标准化厂房，构建集约发展平台。放宽准入、降低门槛，促进民营经济健康发展。完善工业发展政策措施，重点向中小微企业倾斜，不断增强经济发展活力。

　　促进园区建设发展。推动市经开区、高新区向千亿园区目标迈进，加快宜秀、大观、迎江都市产业园建设，启动国家级高新区创建工作。支持桐城开发区按照全省领先、国内一流要求加快发展，实施潜山、宿松、怀宁、枞阳开发区扩容升级。

　　增强自主创新能力。实施“创新安庆”战略，推进科技创新“一招鲜”工程，支持企业研发中心建设，鼓励中船安柴创建国家企业技术中心，扶持非晶软磁、氰衍生物等产业化实体研究院建设。引进省内外大院大所来宜建设大学产业园，支持安师院等市内院校开展产学研合作。实施人才优先发展战略，大力引进高层次创新创业团队，营造人才脱颖而出、各尽其才的制度环境。新增国家高新技术企业40家以上、发明专利申请量1300件以上，全社会研发投入14亿元以上，确保高新技术产业增加值增长15%以上。

　　(二)加速发展现代服务业。

　　发展金融业。引进商业银行设立分支机构，加强基层网点建设。支持企业创业板、“新三板”上市，发展股权投资和风险投资基金，扩大融资规模。鼓励融资性担保公司和小额贷款公司做优做强，加强运营监管，清理整顿非融资性投资机构。完善征信体系，优化金融生态。全面完成农村合作金融机构改制组建农村商业银行工作，实现村镇银行县域全覆盖。鼓励金融机构扩大社会融资规模，确保信贷增长全省前列，提高直接融资比重。

　　提升商贸物流业。完善商贸物流业布局规划，引进林安物流等大型企业，壮大配送、快递等行业。完成人民路步行街改造，加快百联购物广场建设，积极引进国内一线品牌入驻我市建设城市综合体项目。加强口岸建设，健全大通关体系。争创国家保税物流中心、国家外贸转型升级示范基地、全省商贸流通综合改革发展试验区。

　　壮大文化旅游业。积极引进省内外具有较强竞争力的企业入驻安庆，整合文化旅游资源，将资源优势转化成产业优势。继续举办黄梅戏展演系列活动。深入实施“师带徒”工程，培养名角，培植精品。支持市区“一日游”产品开发和推广。实施“景区品质提升工程”，支持五千年文博园争创5A旅游景区，建成中国桐城文化博物馆，加快千年宜城记忆广场等项目建设，推进天柱山世界地质公园博物馆项目。确保旅游总收入增长13%以上。

　　培育新业态。积极发展2.5产业。引进全国知名电子商务平台商在我市布局区域性物流基地和配送中心，吸引国内一流运营商来宜建设电子商务产业园。建成“556电子商务园”，支持淘宝网“特色中国·安庆馆”开业运营。大力推进住宅产业化。启动市会展中心规划论证工作。推进养老、医疗、康复联动，发展健康养老业。

　　(三)全力推动县域经济发展。

　　增强县城和中心镇承载力。按照卫星城市、县级中等城市、特色小城市定位，支持桐城融入合肥经济圈，推进怀宁、枞阳与中心城市同城化发展，继续推动潜山“撤县建市”。完善城镇水、电、路、气设施，增强公共交通、垃圾污水处理能力，健全“文教卫体安”体系，促进人口城镇化。开工建设太湖220千伏等7个输变电工程，推进桐城抽水蓄能电站项目前期工作。坚持产业立县，统筹发展技术、资本密集型和劳动密集型产业，重视就业容量大的服务业发展，培育支柱产业，打造产业集群，支持各县(市)错位竞争、彰显特色。

　　发展现代农业。落实强农惠农政策，促进粮食增产，维护粮食安全。启动实施“5211”工程，推进优质粮油、高效园艺、特色水产、有机畜禽、生态林业五个“百万”工程，加快国家农业科技园区发展，规划建设农产品大市场，扶持十大龙头企业、十大名牌农产品。推进规模经营，发展“一村一品”。新增农民专业合作社300家以上、家庭农场1000个以上，培训职业农民6000人。

　　推进美好乡村建设。抓好84个中心村建设，发展乡村旅游，强化产业支撑，壮大村级集体经济。加强基础设施和公共服务设施建设。力争改造农村危房3万户，改造县乡公路220公里、农村危桥146座。完成40个乡镇农村清洁工程，实现全覆盖。实施城乡环境治理三年行动计划，当年基本消除“三线三边”垃圾、杂物污染问题。

　　加强水利基础设施建设。开工建设下浒山水库，启动大沙河下游治理工程，推进引江济淮、皖河流域综合治理等项目前期工作。继续抓好小农水重点县建设，完成38个小型病险水库除险加固、12条中小河流整治，治理180平方公里水土流失，解决25万农村人口饮水安全问题。建成44万亩高标准基本农田。推进小型水利工程改造，实施排灌泵站三年改造提升规划，当年完成10个。开工破罡湖闸站改扩建工程。

　　做好扶贫开发工作。抓住省级领导牵头联系和省直部门对口扶贫机遇，深入推进大别山革命老区振兴规划实施，加强基础设施建设，加大人力和自然资源开发、金融保障等政策扶持力度，突出抓好教育扶贫，不断增强内生发展动力。实施150个“整村推进”工程，力争脱贫12万人以上。

　　(四)不断加强中心城市建设管理。顺应新型工业化、城镇化进程加快趋势，必须切实增强中心城市综合承载力和带动力，建设宜居宜业的良好环境。

　　加强城市规划建设。优化城市空间布局，将山边、江边、湖边、河边划为有序开发区域，老城区作为限制开发区域，新城区作为鼓励开发区域，东部新城区作为近期重点开发区域。加强控制性详规修编工作，完成15个专项规划编制。按照“老城改造、新城配套”思路，实施50个市政基础设施重点项目，当年完成投资66.3亿元。大力建设广场、游园、绿地，配套公厕、健身、文化、垃圾收集转运设施。改造棚户区100万平方米以上、老旧小区26.3万平方米。着力“打通主干道、畅通微循环”，实施“一环两横三纵”规划。加快建设市区快速通道，开工建设外环北路、环城东路，完成集贤南路、迎宾东路、华中西路等道路黑色化改造，加快推进中兴大道公铁立交工程建设，着手编制城市轨道交通规划。整治背街后巷104个，力争全面完成改造任务。基本完成康熙河(新河)治理工程，启动景观带建设。完成市区24条道路绿化提升工程。开工建设保障性住房2.9万套、建成1.6万套，市区建成还建房109万平方米。推进房地产业持续健康发展。加快“智慧城市”建设。

　　提升城市管理水平。落实“门前三包”责任，深化交通秩序治理，重罚违法违规行为，巩固文明创建成果。开展城市管理五大专项整治行动，拆除占道经营设施，清运城郊结合部垃圾，处罚乱扔乱停乱放，整治车辆超载泼撒，规范户外广告和店招。健全集约节约用地机制，完善征地制度。巩固土地“三项清理”成果，建立清理闲置低效利用土地长效机制，提高土地利用效率。

　　(五)加快建设综合交通体系。建设区域性中心城市，必须抢抓机遇，努力建设成为全省乃至全国重要的综合交通枢纽。

　　着力推进铁路建设，加快建设宁安城际铁路安庆站站房和站前广场。推动合安九客专前期工作实质性进展，积极为开工建设做好准备。推进阜景铁路、湖北随州—安庆铁路、南京—安庆—武汉疏港铁路前期工作。启动月山综合交通枢纽规划建设。着力推进公路建设，加快岳武高速、望东长江大桥及北岸连接线建设，推进合安高速拓宽，开工安庆高速出入口北移工程，争取3个县(市)新增高速公路出入口，推进北沿江高速、济祁高速、岳武高速东延线项目前期工作。加快建设安庆—桐城、安庆—望江、安庆—枞阳、潜山野寨—桃花铺一级公路。实施397公里国省道改造提升工程，当年完成150公里以上。着力提升水运能力，加强岸线资源保护利用，完成石门湖航道疏浚工程，启动内河航道升级三年行动计划。着手安庆港区布局调整，开展皖河深水港区、码头集中区规划建设，推进市区码头外迁。着力改善空运条件，完成安庆机场跑道盖被工程，实施航站楼扩容改造，推动新机场前期工作实质性进展。着力优化管道运输布局，启动石化输油管道迁移规划论证工作。

　　(六)扎实抓好生态建设和环境保护。绿水青山就是金山银山，改善环境也是改善民生。

　　加快“创模”步伐。推进菱湖风景区水环境综合整治，启动市区备用水源建设，加快市一水厂异地扩建。启动石化新装置安全卫生防护距离隔离带建设。实施大气污染防治行动计划，开展PM2.5监测，推进秸秆禁烧工作，加强工业废气、机动车尾气、建筑扬尘、锅炉烟尘治理，加快“五小”企业关停并转，推进公交车、出租车天然气利用，建设“气化安庆”。实行“黄标车”区域限行。新建市区50公里污水管网、60公里雨水管网，建成北部新城污水处理厂，完成城东污水处理厂扩建工程，规划建设石塘湖截污工程，建成石化催化装置脱硫和3号、6号锅炉脱硫脱硝工程，谋划市固废处置中心项目。推进每个县新增2万吨/日污水处理能力，支持潜山、枞阳、怀宁争创省级生态县。推进建筑、交通运输、商业和公共机构等领域节能工作。

　　深入推进“创森”工作。完成省定20万亩森林增长提质工程任务，建成100公里省级森林长廊示范段，抓好退耕还林、血防造林、长江生态防护林建设。加强资源保护、森林防火、松材线虫病防控。支持太湖争创省级森林城市，创建森林城镇10个，确保成功创建国家森林城市。

　　(七)进一步加强社会建设和改善民生。

　　提升民生工程。坚持民生优先，加强政策支持，实施好33项民生工程。与此同时，切实做好与群众密切相关的民生事项，开展老人日间照料服务，落实特教学生生活补助，免除市区义务教育阶段教科书费，实行市区重性精神病患者住院补助。深化菜市场、早餐摊点、夜市排档和豆腐作坊整治。加快公交枢纽站建设，开通安庆至海口公交，推进城乡公交一体化。

　　做好就业和社会保障工作。加强创业就业服务平台建设，做好高校毕业生、城镇就业困难人员和农民工就业。发挥职业教育联盟作用，服务重点企业用工。扩大社会保险参保面，实施医疗大病补充保险。继续提高城乡低保标准，确保全面达到全省平均水平。推进住房公积金归集扩面工作。保持物价基本稳定。

　　加快社会事业发展。按照体现体育精神、带动全民健身要求，承办好省运会、残运会。全面实施国家公共文化服务体系示范区建设。加强文物工作，保护历史文化遗存。开工建设市博物馆、文化馆，规划市图书馆、美术馆。以国家中小学教育质量综合评价改革实验区建设为契机，提升教育质量，打造教育名市。大力推进学前教育全覆盖、义务教育均衡化、高中教育优质化、职业教育规模化，提升高等教育服务地方发展水平。探索实行义务教育阶段教师跨校流动。加快建设桐城师专新校区、安庆一中龙山校区。稳定扩大新农合参合面，进一步提高保障水平。完善医疗卫生服务和基本药物供应保障体系，全面推进县级公立医院改革和医药分开。加强血吸虫等地方病防治。推进市立医院、第一人民医院、中医院新院区建设。扩大计划生育“新三为主”试点，落实国家计划生育新政策。深化殡葬改革，推进“青山白化”整治，不再新批经营性墓园，加大公益性墓园建设力度，开工建设烈士陵园，加快推进回民公墓规划建设。

　　创新社会治理。出台“1+4”社会服务政策，加快社会组织培育和社会工作人才培养，加强社会服务平台建设，加大政府购买服务力度，逐步提高社区工作人员待遇。完成第九届村民委员会换届。开展“安全安庆建设年”活动，严防重特大事故发生。全面开展第三次全国经济普查工作。认真实施“六五”普法规划。大力推进社区网格化建设，加强人民调解工作，支持法律援助，开展社区矫正。实行市县两级联合接访，集中化解群众反映突出问题。推进科技强警，实施“天网工程”，增强社会治安防控能力，严厉打击违法犯罪活动。健全应急管理体系，常态化开展应急演练。抓好地质灾害防治。充分发挥工会、共青团、妇联等人民团体作用。做好科普、地震、气象、档案、地方志、外事、侨务、民族、宗教、涉台、老龄、红十字、关心下一代等工作。支持驻宜部队建设，加强国防教育、国防动员、人民防空和民兵预备役工作，深入开展“双拥”共建活动。

　　(八)全面深化改革和扩大开放。

　　深化行政体制改革。完成工商、质监和食品药品监管体制改革，推进政府机构改革，整合市直部门内设机构，积极稳妥推进事业单位分类改革。深化行政审批制度改革，推进政务服务标准化建设，全面公开审批项目，逐步取消非行政许可事项，精简调整30%以上。实施开发区管理体制和运行机制改革，强化绩效管理。深化城市管理体制改革，建设数字化管理系统，推进重心下移，强化区级政府城市管理职能。全面推进城区社会管理体制改革，整合优化街道、社区、派出所布局。继续深化建设管理“六分开”、公益性公用工程“四到位”。深入开展土地推介活动。不断推进招投标和公共资源交易改革。深化国资管理体制改革。完善管理体制，理顺监管机制，规范资产处置，确保国有资产保值增值。大力推动国有资本进入公益性领域、逐步退出竞争性领域，积极创办污水处理、供气、公交等公益性国有企业，推动华茂整体上市。深化财税体制改革。按照财权事权匹配原则，改革市区财政体制。改进预算管理制度，实施全口径预决算。深化农村改革。加快推进农业经营体制、农村产权制度、城乡发展一体化体制机制改革，开展农村集体经营性建设用地流转、集体土地确权颁证工作。支持宿松、潜山开展省级农村综合改革示范试点。

　　全方位招商引资。大力推进精准招商，举办北京、上海、广州等招商活动，积极拓展境外招商。主攻央企、知名民企和外企，引进成长性强的高新技术企业和行业龙头企业。全年引进亿元以上项目不低于250个，其中10亿元以上不低于30个。扩大对外贸易。推动出口结构升级，鼓励扩大高新技术设备和关键零部件进口，引导外资更多投向高端制造、节能环保等领域。确保外贸进出口增长20%以上。

　　三、切实加强政府自身建设

　　新的一年，发展任务繁重，政府责任重大。我们必须适应形势变化、顺应群众期待，切实增强进取意识、机遇意识和责任意识，以开放的思路、创新的举措、务实的作风，奋力拼搏，扎实苦干，努力建设为民务实清廉政府。

　　坚持执政为民。认真开展群众路线教育实践活动，努力“接地气、增底气”。坚持每月市政府领导公开接访，每月安排市政府负责人通过“1584”政风行风热线与市民互动，每月开展电视“百姓问政”。

　　坚持依法行政。自觉接受市人大及其常委会的监督、政协的民主监督，以及舆论监督和社会监督。完善政府工作规则，坚持法律顾问制度，健全依法民主科学决策机制，推行行政处罚“群众公议”。深化政务公开，办好政府网站，实行决策、执行、结果全程公开。加强政府新闻发布，大力宣传政策措施，做到应知尽知。推进财政预决算、土地招拍挂公开透明。

　　坚持务实高效。加强调查研究。加快转变政府职能，减少审批，减少环节。开短会、讲短话、行短文。实行政策绩效评估制度。继续坚持每两个月集中开工一批工业项目，每两个月举办一次银企对接活动。继续推行一线工作法、推土机式工作法、现场解决问题工作法。强化目标考核，严格问责问效。

　　坚持开放创新。增强自信，大力创新突破。完善体制机制，让专业的人干专业的事，正确的事正确地干。先发地区成功成型经验，大胆借鉴应用。积极争创全省乃至全国示范试点，单项工作创一流，整体工作上台阶。

　　坚持清正廉洁。严格落实党风廉政建设责任制，加强廉政风险防控。强化行政监察和审计监督，坚决惩治吃拿卡要行为，严肃查处违法违纪案件。认真执行《党政机关厉行节约反对浪费条例》，压缩“三公”经费支出。健全激励约束机制，着力打造会干事、好共事、干成事、不出事的公务员队伍。

　　各位代表、各位委员、各位同志，目标已经明确，任务催人奋进。让我们在中共安庆市委的坚强领导下，稳中求进，改革创新，有力有效推进发展，全心全意改善民生，为加快建设经济强市、文化强市、生态强市而努力奋斗！
